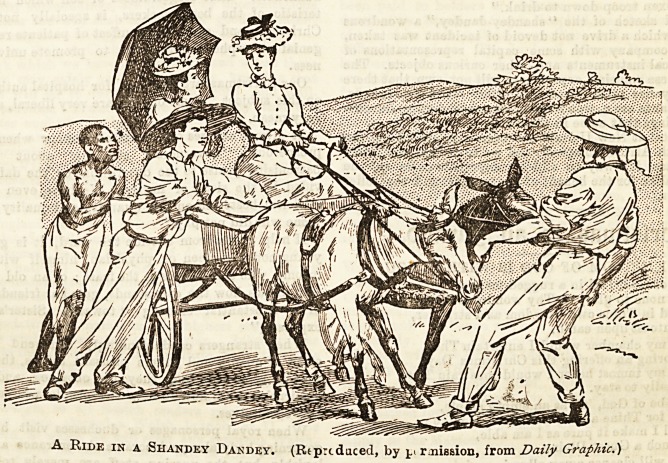# The Hospital Nursing Supplement

**Published:** 1892-12-24

**Authors:** 


### "The Hospital" Nursing Mirror.

Being the Extra Nursing Supplement of "The Hospital" Newspaper.

[Contributions for this Supplement should be addressed to the Editor, The Hospital, 140, Strand, London, W.C., and should have the word "Nursing" plainly written in left-hand top corner of the envelope.]

### En Passant.

A HAPPY CHRISTMAS.—At home or abroad we wish our readers, correspondents, and friends a happy peaceful Christmas! To some it will be an anxious day of work and watching, while to others it will be a "heavy" day because amusements and festivities are not arranged without somebody working hard. But everybody really likes Christmas however it comes, and nearly everyone of us can do a small service of some kind on that day and put a little cheeriness into a life less happy than our own, and though all of us may not feel merry we can at least feel "glad" on the one day in the year when everyone strives to put away ill-feeling and participate in the "goodwill" of the season.

TORONTO GENERAL HOSPITAL.—The presentation of certificates and medals to the successful nurses of the training school of this hospital was held in the theatre, and the ceremony was performed by his Honour the Lieutenant-Governor and Mrs. Kirkpatrick. Miss Mary Snively, the Superintendent, gave an excellent report of the progress of the school, and Dr. McFarlane spoke a few words to the graduating class. Twenty-seven nurses received their certificates.

CUMBERLAND INFIRMARY.—The new nurses' home at this infirmary is to be built at a cost of two thousand two hundred pounds, which leaves three hundred pounds in the hands of the treasurer. Although the furniture will be moved from the old home, there will be carpets to buy, and many other large and necessary items to provide for. But as the new home for the nurses was one of good Bishop Goodwin's scheme, everybody will, we should imagine, be glad to give their mite and push forward till it is completed.

AN APOLOGY.—We hear that the correspondent of the *St. James's Gazette* has called upon Miss Hicks and offered her an ample apology for the inaccuracies of the "interview" which appeared in that journal some week or two ago. The following is an extract from the *St. James' Gazette* of 14th inst.: "Miss Philippa Hicks requests us to state, with regard to the interview with her we published the other day, that one or two slight inaccuracies relating to her statements about the private nurses and her knowledge of their residences were included in our article."

ST. LAWRENCE'S CATHOLIC HOME.—The first report of the General Committee of this Home shows that the many difficulties which attended the installation of the Queen's Jubilee Institute for District Nursing in Ireland have really been overcome. There are now two chief training schools in Dublin, one at St. Patrick's, where, under Miss Howell, the Protestant nurses are trained, and the St. Lawrence's Home in Mary Street, which, under the superintendence of Miss St. Clair, who was trained in the Nightingale School, is devoted to the Catholic nurses, who now number three. The Lady Superintendent in Chief of the Institute in Ireland is Miss Dunn, by whose kindness and tact many rough places have beem smoothed; and the movement also owes much to Lady O'Hagan, who has been a most valuable member of the Committee. The Dublin branch seems to have succeeded well in working in with existing charities, showing how much co-operation is possible between various workers and agencies. Altogether the report is a most interesting one, and we can only hope that the success of this first year will be repeated.

NURSING SCHEME AT GATESHEAD.—It has now been definitely decided to found a nursing association for the sick poor at Gateshead-on-Tyne. The successful initiation of the scheme is in no small measure due to the sympathetic and practical exertions of the Marchioness of Londonderry, who by her warm advocacy of the needs of such an institution has shown how deeply she has the cause at heart. A meeting will shortly be held for the purpose of deciding on the constitution of the association, and electing a council. Mr. C. F. Lloyd has been appointed Secretary to the institution. The success which has crowned the launching of a similar scheme at Stockton-on-Tees fifteen months ago augurs well for the new one.

"FUSS."—"Mr. C. A. Dawson, Local Government Board Inspector for the North-Eastern District, attended the last meeting of the Auckland Board of Guardians, and reported that on visiting the workhouse he had found an inmate suffering from a broken collar-bone, the result of an accident, which had occurred four days before. Neither the doctor nor the nurse were aware of it. Mr. Dawson pointed out the need of more efficient nursing staff, and the appointment of a trained nurse. The guardians were of opinion that seeing the man had not made any complaint, no blame attached to the officers. One guardian said the question of trained nurses was largely gone into at the last Gilsland Poor Law Conference, and it was his opinion that a great deal of the fuss made about the matter was made by those interested in such appointments." We could wish that the necessity for such facts as these finding their way into the Press were less frequent; it is incredible almost that cruelty such as this is tolerated, and that "no blame attaches." The least mistake or oversight in a voluntarily-supported hospital and it is blazoned forth to the whole world. People are clamouring for rate-supported hospitals—may they be far off if the management of them is equal to that of some of our workhouses.

SHORT ITEMS.—Over two thousand pounds has been handed over to the Lincoln Nurses' Institute by the Committee of the bazaar lately held in its aid. A probationer-nurse is to be engaged for Fulwood Workhouse, who is to work under the Superintendent Nurse.—The two district nurses at Portsmouth, Miss Day and Miss Lawden, are leaving to take up private nursing.—The Blairgowrie Association will be known as the Blairgowrie and Rattray District Nursing Association.—Nurse Salter has been appointed district nurse in Ashburton, in place of Nurse Pike.—The Campbeltown Nursing Society is two years old, and during the past year the nurse has paid 3,456 visits.—The Christmas number of *Sylvia's Home Journal* will interest nurses. The first part contains portraits and biographies of well-known women, amongst them Miss Nightingale, Sister Dora, Sister Emma, Dr. Garrett Anderson, and Dr. Jane Walker.—The last report of the district branch of the Bradford Nurses' Institution shows that 34 cases were continued from October to November, 13 entered during the latter month, 47 attended, 10 discharged, and 37 continued to December. The visits paid by the nurses numbered 632.—The Sister-in-Charge of the St. Alban's Diocesan Institution of Nurses has been studying the Q.V.J.I.N. system of district nursing in London. She is so impressed with its thorough work that affiliation is contemplated.—The Douglas District Nursing is now worked from the Hospital. The nurses take it in turn two or three months at a time.—The Treasurer of the Hammersmith and Fulham District Nursing Association is appealing for help.—Nurse Long, who was dismissed from her post in the Peterborough District Nursing Association because she recently joined the Church of Rome, has obtained, through the Royal Jubilee Nursing Institute, a situation at Camberwell.

### Where Some of Our Nurses are Spending Christmas.

### NURSING IN THE LAND OF OPHIR.

It has been a great pleasure to us to get the monthly edition of The Hospital with tolerable regularity, and so find ourselves somewhat in touch with the nursing world at home; and perhaps some of your readers who have been interested in Sister Lucy Sleeman's account of our walk up, which appeared in a *Times* of last December, may like to hear how we have got on since, and what sort of life is being led by nurses in the ancient kingdom of the Queen of Sheba. Well, our first experience was the most intolerable, and might really have hopelessly discouraged women far more resourceful than we can pretend to be.

For weeks after our arrival we were shut up in a tiny hut, which the manager of a mining camp lent us, for, as we were not expected, it being supposed that women could not possibly walk, no arrangements were made for housing us. A new country is filled with the most extraordinary and contradictory rumours, and till people appear in the flesh it would in most cases be extremely foolish to prepare for their arrival. We lived in this hut without pens, ink, paper, or books, with so few candles, that one just burnt a candle-end to undress by; and after about thirteen hours of darkness there came the long, empty day. As our boys had deserted us *en route*, we had no luggage with us, and our shoes being in rags, and kept on our feet with bandages, we couldn't explore the country. Fortunately the owner of the camp had about 100 hens and chickens, and we devoted ourselves to looking after these creatures, watching their fights and tempers, and finding likenesses to our acquaintances amongst them.

In spite, then, of the kindness of our host, it may be imagined with what joy we moved over to some huts which had been built for a camp hospital; whilst some hospital huts were to be put up as soon as possible between our huts and the lines. We moved over at the end of August, and towards the end of September our first patient appeared. There was no hut ready for him, so he brought a tent. He was the last sort of patient one would expect out here, being quite an old phthisis case. However, he looked as if he might be patched up for a bit. My diary says: "No one knows where the patient's food is to come from. At present we have no power to order anything. It is finally settled that 'something' is to come from the camp. 'Something' came in the shape of rather stale tinned lobster. We lose patience at once and for ever, take up the responsibility, and unearth some Liebig, Marjena, and an old hen, and with this, two tin mugs, and an iron spoon we start the hospital."

"October 4th (diary).—Patient worse; fear he is beyond patching. Night work terrible. Days broiling, nights wild and stormy; hyenas wander round with unearthly cries. Tent too small to sit in. Sister Lucy and I rush up and down the hill with a lantern, in mortal and, no doubt, foolish terror, for everyone says that hyenas are too cowardly to be harmful. Still, wild beasts are very unexpected in their ways. For instance, a week or so ago poor Mr. T. was dragged from under his waggon by a lion, and devoured by the lion and lioness, though lions are said never to touch a man if bullocks are near or if a native is to be had, and Mr. T. had a native under the waggon and his oxen close by. We knew him; he came to see us and sent us venison just before he was killed. Poor fellow, his cries were heard for fully ten minutes after he was taken. All this happened five miles away, but as lions travel 30 miles in a night, I can't be sure that one of these won't come round the corner just as we are going up or down.

Saturday, October 10th.—Our first patient is dead. We have four more in the hospital hut, not very bad—three remittent fevers and one dysentery. Mr. Rhodes is here, also the company's administrator, Dr. Jameson; Dr. Jameson very kind. The company is to feed us and the patients, who pay so much a day—if they have any money! In this way we cost the mission absolutely nothing, and I can get proper food for the sick. No more tinned lobster!" Mr. Rhodes left the next day. He came to see us first, and gave us £150 for the hospital. "We are all," says the diary, "camp and hospital and township, to move five miles off; our huts and the hospital to be enclosed by a wild beast-proof fence."

These new huts were not ready till December 13th. Meanwhile we had patients almost constantly in the one hospital hut. It was a wretched place. The rains began in November, and poured into this hut, where the patients slept on beds made of sticks and mouldy grass. Our own huts leaked everywhere. We used to sit in a heap just in the middle of the hut with literally streams of muddy water running down on every side.

On December 13th we moved over to New Umtali. The hospital accommodation was now fairly good, it consisted of a long barn-like building unequally divided into a large ward, a smaller one, and a small operation room. It was totally unfurnished. We were resolved to move heaven and earth rather than have any more grass and stick beds, and after great difficulties, after nearly having to tramp over the mountains to Massikesse, where we heard the Portugese had a store of canvas, we succeeded in getting a waggon sail, and having tolerably comfortable canvas stretchers put up. In the intervals of ward-work we became carpenters, and Sister Lucy having a talent for this sort of work, we were able to turn some packing cases into little bed tables, trays, and other necessaries. It was a long time before we had any blankets, cups, or plates. Each patient brought in his own blankets and mug, and generally clamoured to keep on all his clothes, especially if his temperature was somewhere between 104 degrees and 106 degrees; this being one of the local superstitions. Apropos of the canvas stretchers, the difficulty of moving and changing a helpless patient on one of them passes belief, and nearly breaks one's back, but on the other hand, we have never had the slightest trouble with any patient's back, and we have had several cases of complete unconsciousness for days, combined with a high temperature—one case being complicated by a spinal injury. We had neither spirits of wine nor powder. Brandy cost 30s. a bottle, was often difficult to get, and therefore could only have been used in an extreme case. Our first patient arrived in September, 1891; and now, in September, 1892, we have had seventy-six cases; out of these we have lost eight. People at home who read these numbers will think we have been leading an idle life; but they must remember that besides the nursing the whole of the cooking, and for some time the baking, was on our shoulders. The hospital servants are wild boys, who come in from a kraal, and if they don't run away at the end of a week, almost always leave when their month is over, and then you begin all over again with boys who understand nothing. The slighter cases of remittent fever do not require a night nurse; but when we had bad cases in hospital we found it less tiring to take it in turns to remain on duty for twenty-four hours than to divide night and day duty between us, as in the latter case, between the cooking, cleaning, and ward work, the nurse on day duty was hard at work the whole day, and this in an exhausting tropical climate.

However, our present doctor, Dr. Johnston, a Bart.'s man, has put an end to this arrangement, for he and his dispenser take most of the night work. Still, what we have found the most trying part of life here is not the average daily amount of work, but its continuity. Sometimes for six weeks or two months we have not been able to get away from the hospital for an afternoon. Between January 30th and April 30th we got free for one day, and went to a picnic; we had only one convalescent in hospital, of whom the Dispenser kindly took charge. The very next day a bad case was brought in. In a country like this it is impossible for one woman to wander about alone, and it is the fact of there being only two of us that ties us so much. It must always be remembered too that though this climate is very good, as tropical climates go, yet it is a most enervating one. With the glass at 95 degrees in the shade, the idea of standing over a hot fire is anything but delightful, yet the inevitable dinner must be produced! Just now we are living in great luxury, for we have a Coolie cook, but it is only a *pro. tem.* arrangement, as we were too much run down to be able to go on without help. And here let me warn all nurses who think of coming out to Africa that it is futile to dream of doing so unless they are not only willing to cook, but thoroughly capable of cooking, and know how to bake bread, to iron, and to milk, as well as how to pad a splint, or put up a fracture. They must be prepared to bear cheerfully with a life of unvarying monotony, which revolves perpetually round the food supply question. I must say, though, that it is rather amusing to watch the process by which a new country is victualled. Each trader brings up as much drink as he can have carried up, throwing in, to make up the loads, a certain number of easily packed tinned foods. At one time there was absolutely nothing to be had in Manica but sardines. The whole population browsed on them. They would have become as oily as Esquimaux, if sardines hadn't suddenly been replaced by an enormous supply of foie-gras, and floods of whiskey. Umtali was only saved from an epidemic of D.T. and dyspepsia by an interval of nothing at all but musty meal, which was consumed with great discretion.

Of course the larger number of cases admitted to hospital, are cases of remittent fever. That which prevails up here does not at first seem at all dangerous, patients being frequently discharged well three or four days after admission. It takes different forms with different people. Some have incessant nausea and vomiting and a slight temperature, not exceeding 103; others without nausea or any marked discomfort have temperatures ranging from 103 deg. to 106 deg. But all forms of this fever seem, like the influenza, to leave a certain languor and prostration quite out of proportion with the duration of the illness.

Though no one, I think, has died of the fever *pur et simple*, those who have repeated attacks of it soon begin to have all sorts of unpleasant complications. Several patients, all young, and some very sober and steady, who had been saturated with fever poison, ended by having attacks of paroxysmal hæmaturia. We lost three cases. It is, I think, not a common disease at home. We expected to have a great many cases of dysentery, but to our surprise we have only had two. It is curious that out of seventy-six cases we should have had five with epileptiform attacks. However, as a rule, there is a great sameness about the cases, and no nurse whose interests were purely professional could make herself happy here. There are very few interesting cases; for weeks nursing resolves itself simply into cooking for the convalescents.

Then there will be a sudden rush, and work enough for four or five nurses. The only amusements are such as we can make for ourselves with the dogs, the monkey, and our donkeys, "Pills" and "Powders."

We felt the absence of books so much that we wrote to Mr. Rhodes, who most kindly sent us the nucleus of a good little medical library; all our own books were lost on the way up. It is not in the spirit of grumbling and discontent that I insist on the monotony of life out here, but because I hear of many English nurses "pining for Africa!" And I have met more than one in Africa who had come out full of illusions and visions of exciting cases and romantic work, and who broke down completely when face to face with the humdrum reality, the unexpected drudgery, and the entire absence of all the usual appliances which simplify a nurse's work at home.

Rose A. Blennerhasset (Sister Aimée).
The Hospital, Umtali, Manicaland.

A Ride in a Shandey Dandey. (Reproduced, by permission, from *Daily Graphic.*)

---

The word Africa is not without unpleasant associations to others besides Mrs. Jellaby's daughter, whose views with regard to the continent are very familiar to readers of Dickens.

No doubt some parts of the country possess serious drawbacks from a health point of view, and many have been the English victims to the climate of Africa.

It comes, therefore, as a pleasant variety to get a cheerful letter from an English nurse, who writes a hopeful and amusing account of her life in a hospital at Umtali, a town in Manicaland. The *Daily Graphic* has published extracts from another epistle, accompanied by clever little sketches, in its issue of the 10th and 12th inst., and they are pleasant and instructive reading—pleasant because they give realistic pictures of the daily life and work, instructive because they reflect the writer's cheerful adaptability to circumstances. There is no attempt at posing as a heroine, no self-praise nor self-pity—only a picture of a nurse's life and an assertion that things are better than might have been expected.

New Umtali is distinctly a small place, for its population never exceeds 230, but over a fifth of the number have been hospital patients during the short term of seven months. But this is not so serious as it sounds, sufferers from slight ailments being admitted to the wards owing to the impossibility of attending to them in their own dwellings, which are so widely scattered. Many of the most serious illnesses are directly due to intemperance. Nursing includes in Umtali not only the administration, but frequently the preparation of patients' diets. The English ladies have native assistants, but these cannot be trusted to do any unsupervised work.

The nurse whose letter we are quoting from says that the climate is exquisite, and the country itself most beautiful. The river Umtali is the great feature of the place, "the enormously tall grass screening off the landscape, with the mountains in the background far off, and yet seeming 'placquées' against the grass, the river running very swiftly over and against the stones, and elsewhere going into placid pools covered with water lilies. Here natives come and wash, and oxen troop down to drink."

A spirited sketch of the "shandey-dandey," a wondrous vehicle, in which a drive not devoid of incident was taken, appears in company with some capital representations of native musical instruments and other curious objects. The writer of these amusing experiences will not own that there is any annoyance to be faced from either reptiles or insects, though the ants are a wonderfully destructive race, devouring most things that come in their way.

The hospital at New Umtali has been established by the British South Africa Company, and we hope that the latter will soon see its way to add a competent cook to the household, alike for the benefit of the patients and the relief of the nurses.

### Christmas to an Invalid.

### THE KINGDOM OF GOD IS WITHIN YOU.

O Babe of God, laid in a rugged manger,
With none to glory in Thy wondrous birth,
Received in Thine own kingdom as a stranger,
An outcast upon earth.

Here in my chamber would I entertain Thee,
And bring an offering this Christmas Day,
Here in my inmost heart I would constrain Thee,
Eternally to stay.

Dear Babe of God, 'tis as an empty stable,
Unfit for Thine abode, this heart of mine;
Yet will I make it pure as I am able,
For such a Guest Divine.

First, I will cleanse away all vain repining,
Which makes me doubt that Thy sweet will is best,
And in its stead Thou, Lord, shalt be reclining
Within my hallowed breast.

Then will I offer Thee my richest treasures,
Too poor, too vile for such a holy Child;
But He the love and pure intention measures,
And not the gift defiled.

Take then what joy I have in earth's poor blessings,
And turn it into gladness at Thy love;
Take my delight in temporal possessings,
And store it up above.

Take what Thou wouldst, dear Lord, in my poor dwelling,
And cast out all thou see'est to offend;
And where Thou markest my proud will rebelling,
Subdue it to Thine end.

Dear holy Child, I pant, I pine to hold Thee,
For ever in the shelter of my heart,
Daily to feel, as closer I enfold Thee,
How beautiful Thou art.

How can I murmur I cannot adore Thee
In the full concourse with the general voice,
When I may here kneel humbly down before Thee,
And in Thy love rejoice?

### Christmas in Hospital.

### A Nurse's View.

Young nurses who are happy enough to possess unbroken family circles are apt to think it somewhat of a hardship to spend their first Christmas away from home in a hospital ward. When the day is over they generally own "Its not half bad, after all, to be in a ward on Christmas day."

Many thoughtful women looking back will say that they like a hospital Christmas, and those of us who are minus a home circle of our own go further still and say we prefer the festivals spent amongst our patients to many other past anniversaries.

We may awake on Christmas morning with a feeling of loneliness and a tendency to sentimental repining, but no nurse worthy of the name encourages such feelings on December 25th. The patients must have a happy day, they must be helped to forget their pains, their poverty, and the monotony of their sordid lives, and so have all the enjoyment which we can put within their reach. And to do our fellow-nurses justice, we must own that the results are generally admirable. That forgetfulness of self which is the characteristic of the best workers, is specially noteworthy at Christmas, and even the gruffest of patients relaxes in the genial atmosphere, and helps to promote universal kindliness.

Our Christmas fare is varied, for hospital authorities differ on this subject. Some officials are very liberal, and some are very mean.

It always arouses our sense of humour when we see the prosperous "governors" strolling about and giving patronising praise to the decorations or the dainty tea provided by the sister or nurses. We have even known free criticism bestowed on the quantity and quality of both, by "one who did not pay."

If help comes from outside the ward, it is generally the young house surgeon or physician, himself with a limited income, who contributes to the feast; or an old probationer, who knows how the case stands, gets her friends to join her in a substantial offering "towards Sister's Christmas expenses."

When strangers condescendingly commend the general effect of plants and others pleasant things, they would do well to remember how they are obtained, and thus gain inspiration as to how to help the people who so cheerfully help themselves.

When royal personages or duchesses visit hospitals, the governing body always decorates the entrance and corridors lavishly, but the nursing staff are merely told they are expected to make their wards look as well as they possibly can.

What does this mean? Merely that Sister or Charge Nurse must buy for herself whatever she requires. There may be exceptions to this unspoken rule, but it is sometimes a great tax on the nursing staff. Those who have private means spend gladly; those who have none spend sadly of their hard earnings.

We often think that the princes and princesses and the dukes and the duchesses would be quite content to come and see us in our work-a-day garb. A clean, clear entrance is far more suggestive of the good work done within than any expanse of red baize and piles of blossoming plants hired from a florist. If some additional adornments are desirable for these festive occasions, why don't the managers purchase something which will remain to beautify the institution permanently, and to benefit directly or indirectly the suffering poor? No one would question such proceedings as these, and, in addition, a little generosity at Christmastide would make the festival less of a tax on the pockets and on the powers of earnest hospital workers.

### Help the Nurses to Help the Sick.

"I WILL honour Christmas in my heart, and try to keep it all the year. I will live in the Past, the Present, and the Future. Th spirits of all three shall strive within me."—*Dickens' Christmas Carol.*

WITH all the raids upon the old-timed customs which some few amongst us seem to think it necessary to indulge in, this great festival of Christmas has held its ground against all comers. We doubt if there is anybody who has not felt its softening influence in some shape or form, or who does not discern the great part Christmas plays in warming men's hearts to one another; it is the great festival of One who gave everything He had, His work, His life, His love, and the opportunity for doing good comes to every one of us at one time or other of our lives; there is some moment when we might forward the alleviation of the suffering ever round us, or the healing of moral and physical sores, which the stoniest-hearted cannot fail to notice. If we have no money we can help in other ways; we can spread the news to those who can afford to give, that there ARE workers in the world who by their living influence are trying to create a new state of things, who, having realised that morality is killed by filthy unhealthy surroundings, are striving by unostentatious example to clear the way that the spark of goodness latent in every man may have an opportunity of developing.

So it is then that at Christmas time we print a list of places of work which we have investigated, and where those who will give may safely send their money, knowing that any offerings will go to further definite, good, and well-thought-out schemes. Truthfully we can testify that our working nurses ought not to be forgotten at this great time of giving; they are some of our most earnest labourers in the field of progress:—

**The Queen Victoria Jubilee Institute.**—First by precedence, because it bears our sovereign's name, we should like to commend thisI nstitute, which was founded by her Majesty, who gave to it the bulk of the subscription raised by the women of England in honour of her jubilee. It has practically consolidated district nursing (on the nursing of the sick poor in their homes), so now instead of disintegrated efforts this work is a national one. The Institute trains district nurses, and so maintains a continuous supply of nurses to any local associations needing them; it helps, by money grants, to establish local institutions, and by working in unison with affiliated associations it maintains the highest standard of nursing. Its work is absolutely unsectarian. Subscriptions may be sent to the Treasurer of the Institute, St. Katherine's Hospital, Regent's Park.

**The Q.V.J.I.N. Branches.**—This Institute has started a central home and training school for Scotland at 29, Castle street, Edinburgh, so all Scotch readers might help that branch; they have started a Welsh branch at 11, The Parade, Cardiff, to which we will refer any generous Welshman; and at Dublin the central home will be found at 101, St. Stephen's Green, so it will be seen that we are right in describing this work as comprehensive. In all these different spots the work is identical, the nurses go to the poorest and most unhappy, tending the sick, showing the worth of healthy, clean homes, and as Miss Nightingale says, "teaching, without seeming to teach, which is the ideal of teaching."

**The Rural Branch Q.V.J.I N.**—This branch has its London office at 12, Buckingham Street, Strand. It is exactly what its name signifies, and it is spreading its helpful work in every direction; far away in the most benighted spots these "rural" nurses may be found, and help for the extension of the work would be most gratefully received.

**The Nurses' Bed.**—This is a bed which we should like to see founded in perpetuity. It is for the use of any nurse who through sickness or poverty is unable to take a needed rest. The bed is at the Brassey Holiday Home, St. Leonard's-on-Sea, and is subscribed for yearly. We should be more than grateful if some kind friend to nurses would help to form a fund so that it would be always secure in future years. Any information will be given gladly by the editor of this paper, to whom subscriptions may be paid.

**The Royal National Pension Fund for Nurses,** 8, King Street, Cheapside.—This Society, now well known to most people, deserves consideration from those of our readers who, from time to time, may have known what comfort a trained and kindly nurse may bring to sick folk. Firmly established, this Fund affords to nurses an absolutely safe means of providing, at the lowest possible cost to themselves, an allowance during incapacity for work caused by sickness or accident, and a certain income for their declining years. Persons wishing to become Governors of the Fund may qualify by election and annual subscriptions of two or five guineas, or by the payment in one sum of £25 or £50 respectively, according to the privileges desired. The work of trained nurses is arduous and exhausting; and it is ill-paid. The public are asked to subscribe generously to the Bonus Fund in order that modest pensions may not fail to reward those whose lives are spent in anxious care and toil. During the past year the fund has continued to progress; the invested funds now amount to over £132,000, of which more than £80,000 have been contributed by the nurses themselves. Over two thousand five hundred annuity policies have been issued. Owing to the lamented death of the late Duke of Clarence, the president, H.R.H. the Princess of Wales did not hold the reception which the nurses look forward to with such keen expectation. Progress has been made during the year in the affiliation of hospitals to the fund, and it is believed that, as the scheme and its advantages become more and more widely known, there will be a large influx of institutions desiring to avail themselves of the great benefits afforded by federation with the fund. Sickness during the past year has been very prevalent among the policy holders; over £350 has been paid to holders of sickness assurance policies. The death of Dr. Steele, of Guy's Hospital, has deprived the fund of one of its most zealous councillors and one who will be difficult to replace. The Junius S. Morgan Benevolent Fund has been actively at work, and many nurses have received, and are daily receiving, benefits from it which they most gratefully acknowledge. The deplorable nature of many of the cases submitted for decision show the great need there was for the establishment of the fund. The benefits of this fund are confined to (a) members of the Pension Fund who may be in distress, and to assist them in keeping up the payments of premiums on any policies they may have taken out in the society, and (b) to grant annuities to Matrons, Sisters, and nurses who, from no fault of their own, may be, or are, unable to provide for themselves after sixty years of age. This Pension Fund is, we are sure, well worthy of the sympathy and active support of all those interested in nurses and their work.

**Bible Woman Nurse,** 2, Adelphi Terrace, Strand, W.C. Hon. Secretary, G. Selfe Leonard.

**East London Nursing Society,** 49, Philpot Street, Commercial Road, is the East-end great district nursing undertaking, and it is one of the very best with which we are acquainted. It began work in 1868, and helped on the cause immensely. This, like most of the other big institutions, is affiliated with the Queen's Institute.

**Holy Cross Society of Trained Nurses,** Ladbroke House, 38, Ladbroke Road, Notting Hill, W. Nursing Home for Paying Patients, for which experienced nurses can be obtained for all kinds of illness. Much good work is done unobtrusively but thoroughly by this excellent society. Apply to the Matron.

**Metropolitan and National Nursing Association,** 23, Bloomsbury Square, W.C.—This Association has been working at district nursing since 1874, and has established branches in nine different localities of London, which are now managed and supported independently by local committees. Mainly to the organised effort of this associations we owe the further development of district nursing in England. It is not only a home for the nurses who go out nursing, but it is also a training school for district nurses; it is the central training school in London for the Queen's Jubilee Institute, with which it works in every way. Those who go among the poor know what these nurses do, and those who would like to know should write to Miss Hughes, the lady superintendent, who is always very glad to give information.

**North London Nursing Association,** 413, Holloway Road, N.—This Association does excellent work in the northern district in sending out nurses to the sick poor.

**Nursing Sisters of St. John the Divine,** 68 and 70, Drayton Gardens, South Kensington, S.W. Hon. Secretary, Lieut.-General J. E. T. Nicolls. Sister Superior, Selina S. Wilson.

**St. Helena Home for Trained Nurses and Paying Patients** 1, Grove End Road, N.W. Secretary, Mr. W. H. Kidson. Lady Superintendent, Miss Robertson.

**St. Mary's Home,** Plaistow.—Here, at "London's End," or "London over the border," is St. Mary's Nurses'

Home, where Sister Katherine and twenty nurses are trying to cope with some of the misery and sickness of the 100,000 people round them. There is no more desolate spot to be found than marshy, uncared-for Plaistow—a place that seems to belong to nobody. A good parcel of grocery for the invalid kitchen, or any offering of any sort will be looked for eagerly at this home.

The Workhouse Nursing Association, 6, Adam Street, Strand, is one of our very best nursing institutions. It has worked a perfect revolution in the workhouse infirmaries, and trained nurses, thanks to its labours, are now found in many where formerly the pauper "helps" reigned supreme. As a pioneer society it has had to indulge in some hard fighting, but now already the era of better things seems to have come; guardians are becoming more humane, and the ratepayer is awakening to his responsibilities. Still, all is not yet achieved, and a great deal more must be done. It is a most interesting work, and on a very small income has achieved an almost incredible amount of reform.

We have not space to enumerate all the good works which are being undertaken by nurses the world over, but what we can do is to ask everybody who sees this appeal to look carefully round them in whatever neighbourhood they may live, and see if they do not find some ministering nurses striving to do good by bringing health and hope to the sick. There is scarcely a spot in this land of ours where a nurse, either in hospital or district home, may not be found, and few, very few, are in the position to say, "we do not need help"—they all need help and interest, for population grows and grows, and the needs grow with it. We can only add our hope that this Christmas may reap in good harvests for good work, and so commend our appeal to a kindly public.

### Appointments.

Tewkesbury Hospital, Gloucester.—Miss Flora M. Prossor, who was trained at the Liverpool Northern Hospital, has been appointed Matron of the Tewkesbury Hospital, Gloucester, and entered on her duties there on November 28th. Miss Prossor was Sister for some time at St. Marylebone Infirmary, and for the last twelve months has held the appointment of Matron of the Zenana Medical College, where her influence and good work have been of much value.

St. John's Hospital, Droitwich.—Miss Lydia Gillam, who has been appointed Matron to the St. John's Brine Bath Hospital, Droitwich, was trained at St. Mary's Hospital, Paddington, and afterward did very excellent district nursing in connection with St. John's House, Worcester, and for the last few winters worked for the Holland Institute, Nice. Miss Gillam's friends wish her long happiness and success in her new work, and congratulate the committee who were so fortunate as to secure her services.

King's County Infirmary.—Nurse Georgina Hartford has been appointed Matron to this institution, and will enter on her duties early in the New Year. There were eleven other candidates for the post. Nurse Hartford was trained in the Ulster Hospital, Belfast, for a year, and had charge of the female surgical wards at Mercers' Hospital, Dublin, for a year. In 1891 she took up private nursing in Brixton and Pimlico, and then went as charge nurse in the South-Western Fever Hospital, London, until last May, since which date she has been private nursing in Dublin. Nurse Hartford holds most satisfactory testimonials from those with whom she has worked, and we sincerely hope that better things are in store for this infirmary, which hitherto has been entirely in untrained hands.

Medical College for Women, 50, Chambers Street, Edinburgh.—Miss Elsie Inglis, a student of this college, and who obtained the triple qualification given by the Royal Colleges of Physicians and Surgeons of Edinburgh and the Faculty of Physicians and Surgeons of Glasgow, at the examinations in July last, has been elected to the post of House Surgeon in the New Hospital for Women in London. There were a large number of applications, including several from the London and Irish schools. Miss Inglis has also gained the first of the special Pattison prizes, given by the Royal Colleges after examinations for anatomical dissections; whilst the second prize was gained by Miss Grace Giffen, also a student from this college. Miss Giffen was further awarded the Bathgate Gold Medal in Materia Medica. Both the Pattison and Bathgate medals are open to male and female students, and the papers are signed by mottoes only, so that until the award is given the examiner is ignorant as to the successful candidate's name.

### Poor Barker.

"No! I'd rather stay here. I don't want any tea."

The tone was too gruff for Christmas Day—anyone would have said so. But poor Barker had only come into hospital a few days before, on the verge of delirium tremens, and even now the whole head was sick, and the whole heart faint. He was lonely too, and said "he hadn't any friends there if he did go down."

"Going down" meant down to the accident ward in the little hospital where he was, the dearest little hospital you have ever seen. The ward had been a good deal pulled about, and didn't suggest accidents now, except perhaps of teacups. A row of beds had been pushed against either wall, and the middle laid with long tables. Each patient might invite a friend to tea, and the sick folk, men or women, and all whom it was possible to move, were gathered together here. How gay it looked!

Poor Barker, however, was not to be tempted. Not till everyone else had finished, at least, and then, by some means or other, we prevailed, and he "came down."

Poor fellow, you should have seen him! Thin, grey, bent, with a dazed look in his eyes, which he seldom raised, and with his hand constantly across his forehead.

After tea the tables were cleared, and the staff gave a concert. It was very much appreciated, and with the help of some carols, and the giving away of gifts all round, the evening really passed very merrily.

Poor Barker! he seemed quite overcome. One or two patients got up to "return thanks," and had their little say; it was so funny, some of it, but he said later: "I would have liked to get up myself and say a few words, but my heart was too full."

It was a fortnight, I daresay, before Barker went out of hospital. By that time he had lost a little of his dejected look, and had grown to know us and trust us better. There never was a more grateful patient.

One day he said to me, "I should never have taken to drink but for her. It was all through her that I came here at all."

I didn't want to seem too curious, so I asked no questions, and left him to tell the tale as he chose.

"She wants me to go to church with her. I dinna want the woman—she's a witch, that's what she is. It isn't money she wants from me—she's all right that way. I dinna want the woman, and I wilna go to church with her."

And then he told me she was coming to see him that day before he left. Would she be let in? Well, I told him where he could speak with her best. I pitied poor Barker.

She came.

A few minutes later a nurse came running, "Barker has taken his visitor into the garden."

This was against all rules; but there, we just pretended not to see. I, myself, could not help peeping at them. What a woman! Big, gaunt, beetle-browed—and sixty? She was a witch! It was raining slightly, and she held an umbrella as heavy-looking as herself. Sometimes they walked together under it; sometimes the witch stalked on, heedless of her lagging companion. I couldn't help thinking he was wishing he could slip away from her for ever. Poor Barker, always his head down; while, by the position of hers and her gestures you could see she was "giving it him."

What would be the end of it?

A few weeks later Barker came up to the hospital with a little gift.

A neighbouring clergyman was calling at the same time, and we told him the tale of poor Barker. The little romance seemed very funny, and we laughed again, little thinking into what a grand design he was going to weave the meagre little fragment of a story. Next day was Sunday. At all the churches they were preaching memorial sermons for the lamented Duke of Clarence.

The vicar gave his text, "The Comfort of the Scriptures." He pointed out how many and fruitless were the ways in which mankind sought comfort. Some in this way, some in that. "It was only yesterday, at the hospital, that I heard the story of a poor man suffering from delirium tremens, who told how he took to drinking to drown the memory of a great vexation."

Far back in the church a little movement attracted our attention. I glanced round, and caught the eye of "Poor Barker," and by his side sat "The Great Vexation." Matron.

## Figures and Tables

**Figure f1:**